# Vulvar myeloid sarcoma as the presenting symptom of acute myeloid leukemia: a case report and literature review of Chinese patients, 1999–2018

**DOI:** 10.1186/s13000-019-0892-3

**Published:** 2019-11-07

**Authors:** Xilin Zhang, Peichen Huang, Zhuo Chen, Xinling Bi, Ying Wang, Jianhua Wu

**Affiliations:** 1 Department of Dermatology, Changhai Hospital, Second Military Medical University, Shanghai, 200433 China; 2 Department of Dermatology, Shanghai Skin Disease Hospital, Shanghai, 200433 China; 3 Department of Dermatology, Shanghai Children’s Medical Central, Shanghai Jiao Tong University School of Medicine, Shanghai, 200127 China

**Keywords:** Myeloid sarcoma, Acute myeloid leukemia, Female genitalia, Vulva, Chemotherapy

## Abstract

**Background:**

Myeloid sarcoma (MS), which represents a rare malignancy that comprises of myeloid blasts occurring at extra-medullary sites, closely correlates with the onset and relapse of acute myeloid leukemia (AML) and other hemopoietic neoplasm. Female genital system is an uncommon location of MS, with the vulvar MS being even rarer that only eight cases have been reported in English-written literature.

**Case presentation:**

A 47-year-old woman presented with chronic ulceration on her vulva for one and a half month. Microscopic examination of incisional biopsy revealed dermal infiltration of myeloid precursor cells, which were positive for MPO, lysozyme, CD43, CD68, CD38 and CD117. Bone marrow flowcytometric analysis showed myeloblast count of 74%, which expressed CD13, CD33, CD117 and HLA-DR. A diagnosis of AML (M2 type) was made and vulvar MS was the earliest symptom. The patient achieved complete remission after chemotherapy with no evidence of recurrence in a 27-month follow-up. We reviewed the literature and identified 54 cases of Chinese patients with gynecological MS between 1999 and 2018, and discovered that in Chinese population, MS most frequently involved uterine cervix followed by the ovary and vulva, and ovarian MS onset much earlier than other sites. Remarkably, vulvar MS exhibited a high rate of concurrent AML and secondary myeloid leukemia within a short time of its occurrence. Despite its limited distribution, MS should be tackled aggressively with chemotherapy followed by allogeneic hematopoietic stem cell transplantation if the appropriate donor is available.

**Conclusions:**

Female genital MS, especially vulvar MS, should be included in the differential diagnosis of gynecological neoplasm, which will facilitate its early diagnosis and prompt management.

## Background

Myeloid sarcoma (MS) represents a rare malignancy that encompasses immature or mature myeloid blasts occurring at any extra-medullary site with normal architectural effacement. It was first described by Burns [1] in 1811 and termed as chloroma by King [2] in 1853 because a subset of MS contains abundant myeloperoxidase (MPO) and turns green upon exposure to oxygen [3, 4]. Dock identified the association of MS with acute leukemia in 1893 [5], and Rappaport referred it as “granulocytic sarcoma” in 1996 for the neoplasm comprises of immature granulocytic cells and resembles a sarcoma [6]. Although other historical names have been used, MS was recommended by world health organization in 2001. MS might be isolated [7, 8], precede [9], coincide with the onset [10] and relapse [11] of AML, as well as correlated with myelodysplastic syndrome (MDS) or myeloproliferative neoplasm (MPN) [12]. The incidence of MS is between 1.1 and 9.1% in patients with AML, MDS or MPN [11, 13]. MS occurs in nearly any sites, and the most common sites include lymphoid tissues, central nervous system, lung, kidney and gastrointestinal tract [14]. Female genital system is a much rarer location that less than a hundred cases have been reported in English-written literature [8, 10, 11]. The frequency of gynecological involvement from high to low was the ovary, cervix, uterus and vulva [10, 15]. Precisely, only 8 MS patients involving the vulva were identified in literature [16]. Here, we report an unusual case of vulvar MS as the initial presentation of AML, and review the literature of Chinese patients with gynecological MS.

## Case presentation

**Fig. 1:**
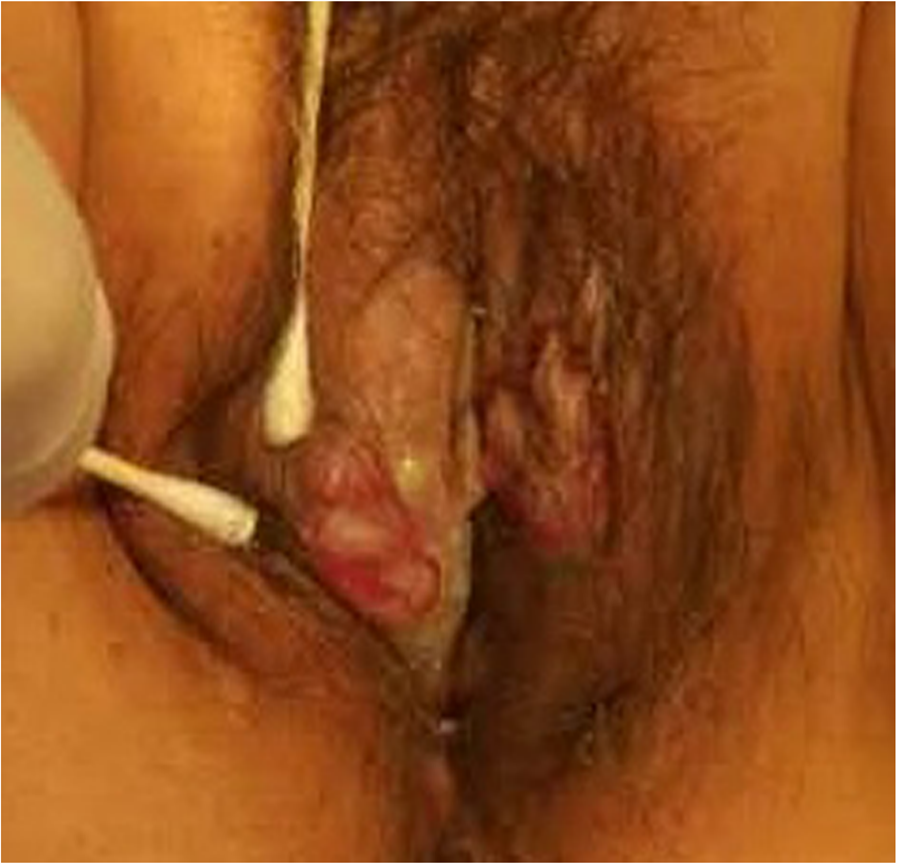
Two large well-demarcated ulcers on bilateral labia majora

**Fig. 2:**
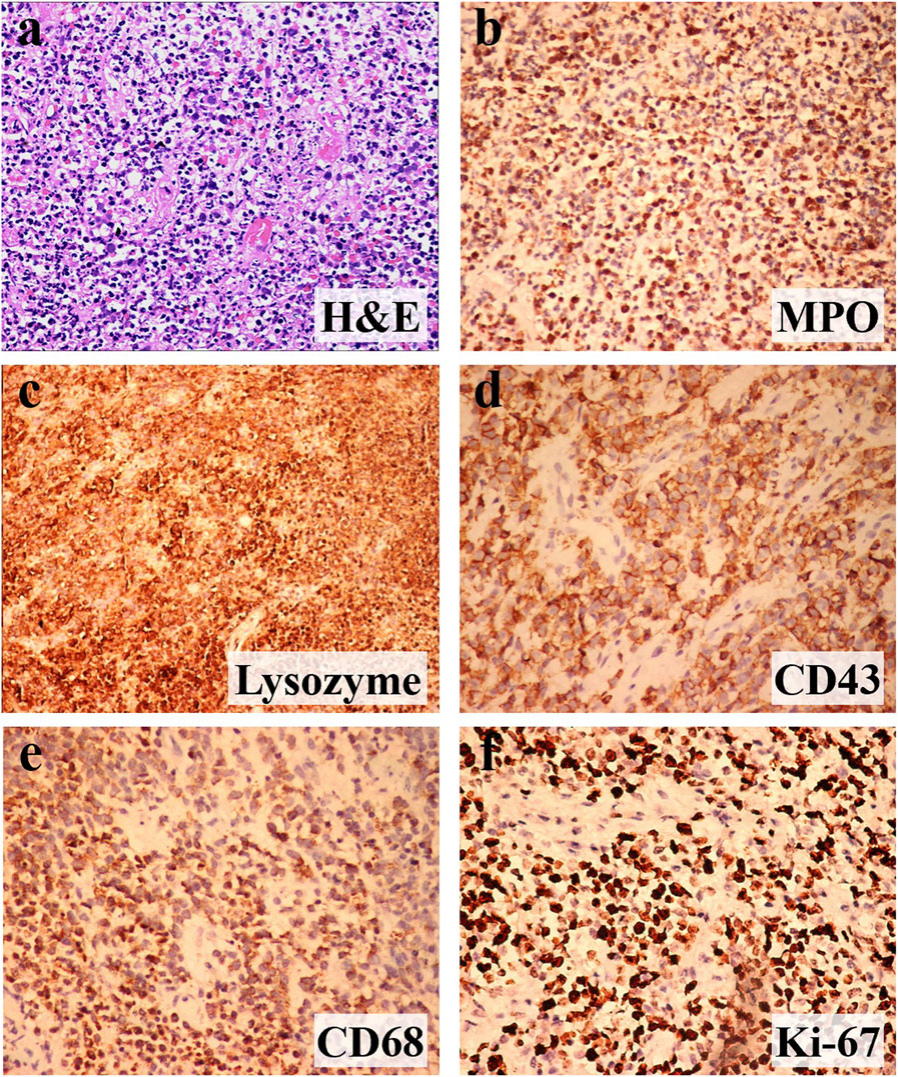
Hematoxylin-eosin and immunohistochemical staining of vulvar ulcers. Diffuse noncohesive sheets of medium-sized myeloid precursor cells in the dermis (**a**); Immunohistochemical staining result is positive for myeloperoxidase (**b**), lysozyme (**c**), CD43 (**d**), CD68 (**e**) and ki-67 (**f**)

A 47-year-old woman presented with fever and chronic ulceration on her vulva for one and a half month in January 2017. The patient had no significant past medical or family history. She had been given levofloxacin and topical douche in another hospital, but the vulvar lesions continued to aggravate. Gynecological evaluation revealed two large well-demarcated ulcers on bilateral labia majora (Fig. 1) without involvement of labia minora and vagina. The patient underwent an incisional biopsy and the cut surface of specimen was grey-white. Microscopically, the dermis was infiltrated with diffuse noncohesive sheets of medium-sized myeloid precursor cells that have large vesicular nuclei, prominent nucleoli, and scarce ill-defined cytoplasm with mild pleomorphism (Fig. 2a). Abundant neutrophils and sparse plasma cells were observed. Immunohistochemistry (IHC) demonstrated positive reactions with MPO (Fig. 2b), lysozyme (Fig. 2c), CD43 (Fig. 2d), CD68 (Fig. 2e), CD38 and CD117, and negative reactions with T-cell markers (CD3, CD5, CD56), B-cell markers (CD20, Bcl-2, Bcl-6) and plasma-cell makers (CD138). Ki-67 was expressed in 80% of the neoplastic cells (Fig. 2f). Therefore, she was diagnosed as MS and admitted to hospital.

On admission, her peripheral blood count showed white blood cells 6.78 × 10^9^/L, hemoglobin 80 g/L, hematocrit 26%, platelets 6.78 × 10^9^/L. Differential blood count was as follows: blasts 71%, unclassifiable cells 16%, neutrophils 24%, lymphocytes 58%, monocytes 2%. Her peripheral blood smear revealed the percentage of leukemic cells was 28%, while the bone marrow (BM) aspirate contained 44.5% leukemic cells. Flowcytometric analysis showed myeloblast count of 74%, which expressed CD13, CD33, CD117 and HLA-DR. Cytogenetic study of the BM discovered a normal 46, XX karyotype. Fluorescence in situ hybridation (FISH) analysis did not detect any common fusion genes in hematologic diseases such as AML, MDS, eosinophilia and acute lymphoblastic leukemia (ALL). Given the results, a diagnosis of AML (M2 type, FAB classification) was made and MS of the vulva was the earliest symptom in this patient.

**Fig. 3:**
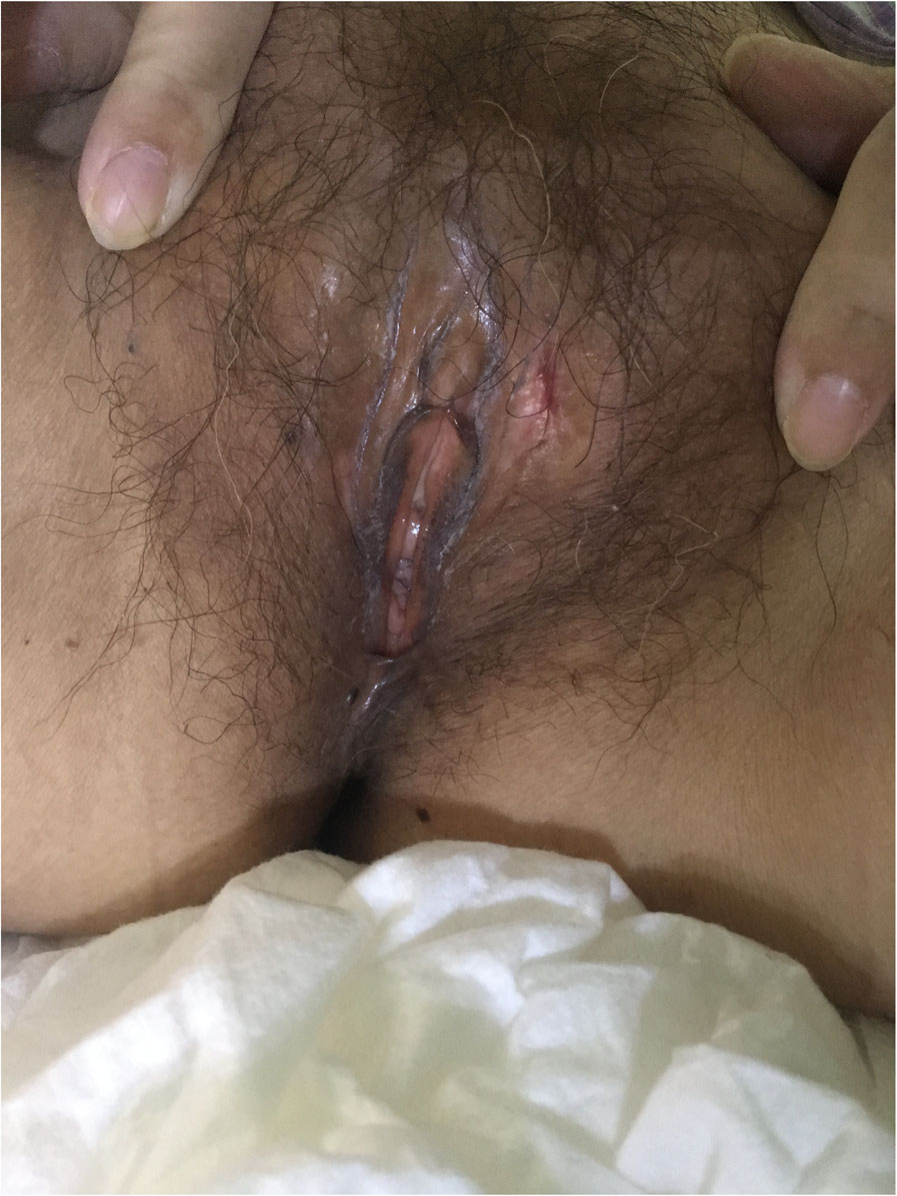
Vulvar ulceration healed after chemotherapy

She subsequently received induction chemotherapy with idarubicin (10 mg/m^2^ for 3 days) and cytarabine (100 mg/m^2^ for 7 days) that achieved complete remission 1 month later with the ratio of minimal residual disease being 0.017%. Meanwhile, the vulvar ulceration healed without other therapy (Fig. 3). In this period, the patient developed upper gastrointestinal bleeding and acute inferior myocardial infarction that recovered after conservative treatment. She then received 5 cycles of intensification therapy (high-dose cytarabine 3 g/m^2^/12 h for 3 days) along with intrathecal injection of methotrexate and cytarabine for 4 times. Neither a family nor unrelated donor for haematopoietic stem cell transplantation (HSCT) had been found. Currently, she remained in complete remission 27 months from the time of diagnosis on follow up.

## Discussion and conclusions

We searched following terms of “genitals and MS” and “genitals and AML” in the PubMed and Chinese literature databases including Wanfang Data (http://www.wanfangdata.com.cn/index.html), VIP Journals (http://qikan.cqvip.com/) and China Knowledge Resource Integrated Database (http://www.cnki.net/). In total, we identified 54 MS cases involving gynecologic tract reported between 1999 and 2018, details of which are summarized in Table 1.

**Table 1:** Reviews of Chinese cases of gynecological myeloid sarcoma

No	Author	Age	Time of genital involvement	Non-systemic involvement	Systemic involvement	AML Type	Treatment	Outcome
Vulva								
1	Huang et al. [17]	78	Initial	None	Simultaneously	NA	Refused	Not stated
2	Yang et al. [18]	27	Initial	None	Simultaneously	M2	CT (DNR + ARA-C)	CR, ANEL 36 mo
3	He et al. [19]	25	Initial	Pelvic LA	Simultaneously	M5	Refused	Not stated
4	Hu et al. [20]	45	Relapse (MDS)	Perirenal	After 1.4 mo of vulvar MS	M5	CT (ARA-C + IDA)	CR, died 3 mo (sepsis)
5	Fang et al. [21]	75	Isolated	Pulmonary SCC	None	NA	CT (HHT + ARA-C) → RT	MS resolved, relapse 10 mo later
6	Our case	47	Initial	None	Simultaneously	M2	CT (ARA-C + IDA, ARA-C) II (ARA-C + MTX)	CR, ANEL 27 mo
Vagina								
7	Li et al. [22]	55	Isolated	None	None	NA	SG → Refused	Died 10 mo
8	Xue et al. [23]	61	Not stated	Regional LA	Not stated	M2	Not stated	Not stated
Uterine cervix								
9	Lu et al. [24]	41	Initial	None	After 19 d of SG	M2	SG → CT	Died 27d (cerebral hemorrhage)
10	Qu et al. [25]	28	Initial	None	Simultaneously	M2b	Not stated	Not stated
11	Zhang et al. [26]	45	Initial	None	Simultaneously	Not stated	CT (DNR + ARA-C)	Not stated
12	Wen et al. [27]	49	Not stated	Not stated	Not stated	NA	RT	Died 2 mo
13	Feng et al. [28]	40	Isolated	None	None	NA	SG	Not stated
14	Gao et al. [29]	34	Isolated	None	None	NA	CT (ARA-C + DNR) → SG → CT (ARA-C + DNR)	ANEL
15	Li et al. [30]	34	Isolated	None	None	NA	CT (DNR + ARA-C) → SG	ANEL
16	Zheng et al. [31]	43	Isolated	None	None	NA	CT (HHT + ARA-C)	ANEL 20 mo
17	Li et al. [22]	46	Isolated	None	None	NA	SG → CT (DNR + ARA-C)	ANEL 8 mo
18	Gu et al. [32]	42	Isolated	None	None	NA	CT (DNR + ARA-C)	ANEL 3 mo
19	Yu et al. [33]	28	Isolated	None	None	NA	CT (IDA + ARA-C)	Died 18 mo
20	Liu et al. [34]	27	Isolated	None	None	NA	SG	ANEL 15 mo
21	Zhang et al. [35]	23	Relapse (AML)	None	After 1 mo of MS	M2	CT (ARA-C + MIT)	CR
22	Liu et al. [36]	46	Secondary	supramaxilla, breast	After 18 mo of MS	Not stated	CT (DNR + ARA-C, HHT + ARA-C, MIT + ARA-C) → pelvic RT	Died 4 mo after cessation of CT
23	Zhu et al. [37]	63	Not stated	Not stated	None	NA	CT → RT	CR
24	Zhu et al. [37]	45	Not stated	Not stated	None	NA	CT → SG	Died
25	Xue et al. [23]	46	Not stated	Head, neck, regional LA	Not stated	NA	Not stated	Not stated
26	Zuo et al. [38]	42	Initial	None	AML during CT	Not stated	SG → CT (DNR + ARA-C)	Died 12 mo
27	Zuo et al. [38]	51	Isolated	None	None	NA	CT → SG → CT (DNR + ARA-C) → gingiva 55 mo later → CT + RT + Allo-HSCT	Alive 91 mo
28	Feng et al. [39]	57	Isolated	None	None	NA	SG → Refused	ANEL 6 mo
29	Liu et al. [40]	50	Not stated	Pelvic LA	Not stated	NA	SG	Not stated
30	Wang et al. [41]	28	Initial	None	After 5 mo of MS	M2a	CT	CR, died 20 mo
Uterine body								
31	Wang et al. [42]	38	Isolated	None	None	NA	SG	Not stated
32	Zhao et al. [43]	33	Isolated	None	None	NA	CT (DNR + ARA-C)	Alive
33	Hou et al. [44]	44	Isolated	None	None	NA	Not stated	Not stated
Ovary								
34	Zhang et al. [45]	27	Initial	None	Simultaneously	M2	CT (DNR + ARA-C)	PR, MS resolved
35	Zheng et al. [46]	26	Isolated	None	None	NA	SG → CT (DNR + ARA-C) → Auto-HSCT	ANEL 1 y after HSCT
36	Yu et al. [47]	35	Isolated	None	None	NA	SG → CT	ANEL 3 y 9 mo
37	Yu et al. [47]	26	Relapse (AML)	None	None	NA	SG	Not stated
38	Yu et al. [47]	24	Isolated	None	None	NA	SG → CT	ANEL 5 mo
39	Zhou et al. [48]	27	Initial	None	After 2 mo of MS	M2	SG → CT (DNR + ARA-C)	Not stated
40	Zhou et al. [49]	36	Isolated	None	None	NA	SG → CT (DNR + ARA-C)	Not stated
41	Zhu et al. [37]	23	Isolated	None	None	NA	CT (ARA-C)	PR
42	Zhou et al. [50]	27	Not stated	Not stated	Not stated	NA	SG	Not stated
43	Pang et al. [51]	23	Isolated	None	None	NA	CT → RT	Died 39 mo
44	Pang et al. [51]	22	Relapse (AML-M3)	None	Not stated	NA	CT	Died 38 mo
45	Zhou et al. [48]	36	Not stated	Lung, small intestine, brain	None	NA	CT (VP-16 + ARA-C)	Died 1 mo (cerebral hemorrhage)
46	Wang et al. [41]	26	Secondary	Small intestine	None	NA	SG → CT	MS resolved, ANEL 15 mo
Multifocal								
47	Zhang et al. [52]	29	Initial (vulva, ovary)	Whole body	Not stated	NA	Refused	Died 1 mo
48	Cheng et al. [53]	37	Initial (uterine cervix, ovary)	Right common iliac lymph nodes	After 6 mo of MS	Not stated	SG → CT (DNR + ARA-C, DNR + ARA-C+ Vm-26) → nasopharyngeal TCL → AML → CT (CTX + ADM + VCR + PED+Vm-26)	Metastasis to chest wall and anterior mediastinum
49	Qu et al. [54]	44	Relapse (AML-M2a) (uterine body, cervix)	None	None	NA	SG → CT	ANEL 1 y
50	Li et al. [55]	43	uterine body, cervix, vagina	Iliac perivascular LAP	None	NA	CT (PTX + PDD) → RT + CT (DNR + ARA-C)	MS resolved, ANEL 6 mo after cessation of CT
51	Wu et al. [56]	25	Uterine cervix, vagina	None	None	NA	CT (IDA + ARA-C)	CR, ANEL 3 mo
52	Wang et al. [57]	43	Uterine cervix, vagina	None	None	NA	CT (DNR + ARA-C, ARA-C, FA + ARA-C+ G-CSF), II (MTX + ARA-C + DXM)	Cervical MS resolved, ANEL 7 mo
53	Long et al. [58]	46	Relapse (AML-M2) (ovary, uterine cervix)	None	1 mo after SG	M2	SG → CT (MTX + VP16 + ARA-C)	ANEL 8 mo
54	Huang et al. [59]	43	Uterine cervix, left appendage	None	None	NA	CT	Died 11 mo
55	Xu et al. [60]	51	Ovary, uterus	Colon, rectum	Not stated	NA	SG, refused	Not stated

*ADM* Adriamycin, *AML* acute myeloid leukemia, *ANEL* alive with no evidence of leukemia, *ARA-C* cytarabine, *CR* complete remission, *CT* chemotherapy, *CTX* Cyclophosphamide, *d* day, *DNR* daunorubicin, *FA* Fludarabine, *HHT* homoharringtonine, *HSCT* hematopoietic stem cell transplantation, *Allo-HSCT* allogeneic HSCT, *Auto-HSCT* autologous HSCT, *IDA* idarubicin, *II* intrathecal injection, *LA* lymphadenopathy, *MIT* Mitoxantrone, *MDS* myelodysplastic syndrome, *mo* month, *NA* not applicable, *PED* Prednisone, *PDD* cisplatin, *PR* partial remission, *PTX* paclitaxel, *RT* radiotherapy, *SCC* squamous cell carcinoma, *SG* surgery, *TCL* T cell lymphoma, *VCR* Vincristine, *VP-16* Etoposide, *y* year

Being a rare entity, isolated MS often poses diagnostic challenge, and immunohistochemical examination is of great importance in the correct diagnosis. As the myeloblasts in MS have an antigen profile resembling that of the blasts and precursor cells in AML, the positivity of myeloperoxidase, CD43, CD68, CD117 and lysozyme help to recognize MS. The most important differential diagnoses include non-Hodgkin lymphoma of the lymphoblastic type, Burkitt’s lymphoma, large-cell lymphoma and small round cell tumors [61]. However, we did not detect any exclusive surface marker of MS involving gynecological tissue.

Our reviewed cohort showed that gynecological MS involved uterine cervix (40%), ovary (23.6%), vulva (10.9%), uterine body (5.5%) and vagina (3.6%) in a most-preferred-to-least-preferred order with around one sixth of cases had multifocal lesions, which differed from previous notion that the most frequently involved genital organ is the ovary followed by the cervix and uterus [10, 15, 62]. The inconsistency might partly result from ethnic diversity. A ‘skip’ phenomenon was also noticed in nearly half of the multifocal MS patients that the myeloid blasts occurred at non-adjacent sites, which is uncommon in other gynecological malignancy.

The age of female-genital MS onset ranged from 22 to 78 years with an average age being 39.2 ± 1.7 years (Table 2), which differed from a predilection of general MS for children [63]. Particularly, MS arising at the ovaries mostly occurred in young adults, which was much earlier than the other single locations (27.5 ± 1.4 vs 43.5 ± 2.3, *P* = 0.0001). Female-genital MS could be asymptomatic (6 cases) or initially presented as mass formation (9 cases), abdominal pain (8 cases), ulceration (1 case), paramenia and vaginal bleeding (25 cases), which was similar to an earlier observation [64]. Remarkably, the onset symptom of all the previously-reported vulvar MS was regional mass with our case distinctively being ulceration.

**Table 2:** Onset age and correlation with AML of reviewed myeloid sarcoma patients

MS site	Onset Age (year)	Without AML	Preceding AML	Coinciding with AML	AML Relapse
Vulva	25–78 (49.5 ± 9.3)	1 (16.7%)	1 (16.7%)	4 (66.7%)	─
Vagina	55–61 (58 ± 3)	1 (100%)	─	─	─
Uterine cervix	23–63 (41.3 ± 2.2)	10 (58.8%)	4 (23.5%)	2 (11.8%)	1 (5.9%)
Uterine body	33–44 (38.3 ± 3.2)	3 (100%)	─	─	─
Ovary	22–36 (27.5 ± 1.4)	8 (66.7%)	1 (8.3%)	1 (8.3%)	2 (16.7%)
Multifocal	25–51 (40.1 ± 2.8)	5 (62.5%)	1 (12.5%)	─	2 (25%)
Total	22–78 (39.2 ± 1.7)	28 (59.6%)	7 (14.9%)	7 (14.9%)	5 (10.6%)

*AML* acute myeloid leukemia

Three fifths of MS patients are not correlated with AML or other hematopoietic disorders, with equally 14.9% cases preceding or coinciding with AML and 10.6% occurring as the first sign of AML relapse (Table 2). While the few cases of vagina and uterine-body MS revealed no linkage with AML, vulvar MS exhibited a notably high rate of concurrent AML and secondary myeloid leukemia in a short time. The interval between the initial diagnosis of MS and systemic disease with medullary involvement ranged from 0.6 to 18 months with a mean value of 5.5 month, in accordance with the formerly-reported 5 to 11 months [65–67]. And, MS heralded AML relapse with or without marrow involvement, and the duration was from 6 to 67 months with a mean value of 33.6 months.

As evidenced from prior observation, FAB subtype M4 and M5 are mostly associated with extra medullary tissue involvement [16]. Unexpectedly, our reviewed cohort displayed a predominance of M2 subtype (10 cases) with the remaining being M5 (2 cases) and M3 (1 case), suggesting that M2 subtype of AML was most inclined to develop MS in the Chinese population. The chromosomal abnormalities of MS include trisomy 4, trisomy 8, trisomy 11, monosomy 7, 16(q)-, 5q- and 20q-, while t (8;21)(q22;q22) and inv [16] (p13;q22) were the most common chromosome rearrangements detected in AML-correlated MS [12, 68]. In our reviewed cases, three occurred t (8;21)(q22;q22), in conformity with the high incidence of t (8;21) in AML-M2 patients with MS [68]. And, one AML-M5 patient had complex chromosomal aberrations of t (1;7)(p22;q36), t (3;21)(q22;q26) and loss of chromosome 16 [20]. Recurrent AML1/ETO fusion genes were identified in two gynecological MS patients, whereas no cytogenetic defect was discovered in five patients.

Despite the local distribution of MS, chemotherapy was more effective than radiation therapy or surgical removal for improving disease-free intervals or survival [69, 70]. Additionally, allogeneic or autologous BM transplantation appeared to increase the odds of prolonged survival [12]. The therapeutic measures taken by the reviewed MS patients were comprised of chemotherapy (16 cases), surgery (3 cases), radiotherapy (1 case) and chemotherapy-combined treatment (19 cases), the majority of which being chemotherapy plus surgery. The chemotherapy regimen, which proved to be helpful even after tumor recurrence, primarily relied on cytarabine (Table 1). Recently, hypomethylating agents including decitabine and 5-azacitdine was considered as another option in the elderly patients [71]. The longest disease-free survival of 91 months (case 27) was achieved in an isolated cervical MS case treated with chemotherapy, surgery and radiotherapy followed by the consolidation of allogeneic HSCT. However, we also noted that surgical removal of isolated cervical MS within 1 month of its onset (case 20, 28) was also successful. In brief, chemotherapy and allogeneic HSCT encompasses an optimal management of MS, and surgery and radiotherapy were ancillary modalities for initial debulking and rapid remission.

Previous evidence suggested that MS generally carries a rather poor prognosis with a 5-year survival rate being about 20%, which were not affected by patient age, gender, MS anatomic site, de novo presentation, history related to AML, histotype, phenotype nor cytogenetic findings [12, 72]. Moreover, the median survival for MS patients with or without AML has been reported to be 6 to 14 months and 36 months, respectively [66]. For the entire reviewed group, the follow-up periods for 18 patients were 3 to 91 months with a mean duration of 18.3 months, whereas 12 patients died of the disease in an average of 13 months (0.9 to 39 months). We further analyzed the survival duration of these gynecological MS patients according to the tumor sites and BM involvement. Vulvar and multifocal MS seemed to have a poorer prognosis, while the medullary involvement might not further worsen their prognosis. Although t (8;21) represents a favorable prognostic factor in traditional AML, it did not indicate a better prognosis in MS [12]. The one AML-M2 patient (case 30) with t (8;21)(q22;q22) received chemotherapy and died 20 months after the diagnosis.

In summary, we herein reported a rare case of vulvar MS and reviewed Chinese MS cases specially involving gynecological system. We discovered that MS most frequently involved uterine cervix followed by the ovary and vulva, and ovarian MS onset much earlier than other sites. Moreover, vulvar MS exhibited a notably high rate of concurrent AML and secondary myeloid leukemia in a short time, which require immediate management. Despite its limited distribution, MS should be tackled aggressively with chemotherapy followed by allogeneic HSCT if the appropriate donor is available. Female genital MS, especially vulvar MS, should be included in the differential diagnosis of gynecological neoplasm, which will facilitate its early diagnosis and prompt management.

## Data Availability

Please contact author for data requests.

## Abbreviations

ADM: Adriamycin
ALL: Acute lymphoblastic leukemia
Allo-HSCT: Allogeneic HSCT
AML: Acute myeloid leukemia
ANEL: Alive with no evidence of leukemia
ARA-C: Cytarabine
Auto-HSCT: Autologous HSCT
BM: Bone marrow
CR: Complete remission
CT: Chemotherapy
CTX: Cyclophosphamide
d: day
DNR: Daunorubicin
FA: Fludarabine
FISH: Fluorescence in situ hybridation
HHT: Homoharringtonine
HSCT: Hematopoietic stem cell transplantation
IDA: Idarubicin
IHC: Immunohistochemistry
II: Intrathecal injection
LA: Lymphadenopathy
MDS: Myelodysplastic syndrome
MIT: Mitoxantrone
mo: month
MPN: Myeloproliferative neoplasm
MPO: Myeloperoxidase
NA: Not applicable
PDD: Cisplatin
PED: Prednisone
PR: Partial remission
PTX: Paclitaxel
RT: Radiotherapy
SCC: Squamous cell carcinoma
SG: Surgery
TCL: T cell lymphoma
VCR: Vincristine
VP-16: Etoposide
y: year

## References

[1] Burns A (1811). Observations of surgical anatomy, head and neck.

[2] King A (1853). A case of chloroma. Monthly J Med.

[3] Schultz J, Shay H, Gruenstein M (1954). The chemistry of experimental chloroma I. Porphyrins and peroxidases. Cancer Res.

[4] Reardon G, Moloney W (1961). Chloroma and related myeloblastic neoplasms. Arch Intern Med.

[5] Dock G (1893). Chloroma and its relation to leukemia. Am J Med Sci.

[6] Rappaport H (1996). Tumors of the hematopoietic system. Atlas of Pathology.

[7] Isonishi S, Ochiai K, Nikaido T, Yano S, Aiba K, Tanaka T (2011). Isolated myeloid sarcoma of the vulva. Clin Ovarian Cancer.

[8] Yu Y, Qin X, Yan S, Wang W, Sun Y, Zhang M (2015). Non-leukemic myeloid sarcoma involving the vulva, vagina, and cervix: a case report and literature review. Onco Targets Ther.

[9] Ersahin C, Omeroglu G, Potkul RK, Salhadar A (2007). Myeloid sarcoma of the vulva as the presenting symptom in a patient with acute myeloid leukemia. Gynecol Oncol.

[10] Policarpio-Nicolas M, Valente P, Aune G, Higgins R (2012). Isolated vaginal myeloid sarcoma in a 16-year-old girl. Ann Diagn Pathol.

[11] Nazer A, Al-Badawi I, Chebbo W, Chaudhri N, El-Gohary G (2012). Myeloid sarcoma of the vulva post-bone marrow transplant presenting as isolated extramedullary relapse in a patient with acute myeloid leukemia. Hematol Oncol Stem Cell Ther.

[12] Pileri S, Ascani S, Cox M, Campidelli C, Bacci F, Piccioli M (2007). Myeloid sarcoma: clinico-pathologic, phenotypic and cytogenetic analysis of 92 adult patients. Leukemia.

[13] Neiman R, Barcos M, Berard C, Bonner H, Mann R, Rydell R (1981). Granulocytic sarcoma: a clinicopathologic study of 61 biopsied cases. Cancer.

[14] Barcos M, Lane W, Gomez G, Han T, Freeman A, Preisler H (1987). An autopsy study of 1206 acute and chronic leukemias (1958 to 1982). Cancer.

[15] Oliva E, Ferry J, Young R, Prat J, Srigley J, Scully R (1997). Granulocytic sarcoma of the female genital tract: a clinicopathologic study of 11 cases. Am J Surg Pathol.

[16] Sahu K, Jain A, Yanamandra U, Varma S, Malhotra P (2016). Myeloid sarcoma of vulva: a short update. Indian J Hematol Blood Transfus.

[17] Huang C, Li J, Huang W (2005). Vulval granulocytic sarcoma: report of a case. Chinese J Pathol.

[18] Yang M (2007). Primary granulocytic sarcoma:a report of four cases and literature review. J Rare Uncommon Dis.

[19] He Y, Li X, Huang Y, Wang D, Hu Y, Huang R (2013). Misdiagnosed myeloid sarcoma of the vulva. Chin Med J (Engl).

[20] Hu S, Chen W, Chen G (2016). Myeloid sarcoma of the vulva as the initial presentation of acute myeloid leukaemia. Br J Dermatol.

[21] Fang S, Wang W, Zhao Y (2017). Granulocytic sarcoma of the vulva: a clinicopathological analysis and review of literature. Cancer Res Clin.

[22] Li D, Yang X, Chang X (2010). Clinicopathological features of myeloid sarcoma involving gynecologic tract. J China Med Univ.

[23] Xue K, Cheng J, Zhang Y, Bai J, Bu C, Bei T (2015). Imaging features of granulocytic sarcoma. Chinese J Med Imaging Technol.

[24] Lu X, Xiao L (1999). Uterine cervical granulocytic sarcoma: report of a case. Chinese J Pathol.

[25] Qu Y, Tang A, Niu A, Xu J, Zhang S (2007). Cervical granulocytic sarcoma as the presenting symptom of M2b-type acute myeloid leukemia: report of a case. Chinese J Hematol.

[26] Zhang S, Zhou X, Zheng Y, Zhang Y, Liu W, Huang S (2007). Granulocytic sarcoma of uterine cervix: a case report and review of literature. Chinese J Diagn Pathol.

[27] Wen Q, Zhu J. Clinical analysis of 13 cases with cervical sarcoma. J Oncol 2007;13(2):129–30.

[28] Feng X, Ying J, Yin C, Li L, Shi S, Zhang H (2008). The diagnosis and differential diagnosis of granulocytic sarcoma. Chin-Ger J Clin Oncol.

[29] Gao J, Zhang G, Shi Y, Song S, Li J, Xu S (2008). Cervical granulocytic sarcoma: report of a case and review of literature. Chinese J Obstet Gynecol.

[30] Li L, Jiang W, Li J, Huang Q, Zhu J, Zhang Y (2008). Uterine cervical granulocytic sarcoma: report of a case. Chinese J Clin Exp Pathol.

[31] Zheng G, Zheng W, Sun J, Yu X, Ye X. Uterine cervical primary granulocytic sarcoma: report of a case. Xiamen: The First National Youth Hematologist Academic Conference; 2009.

[32] Gu T, Shi Q (2011). Clinical pathology analysis of granulocytic sarcoma of uterine cervix. Zhejiang Pract Med.

[33] Yu X, Ye L (2013). Cervical granulocytic sarcoma: report of a case. Chinese J Clin Exp Pathol.

[34] Liu Q, Shi Y, He H, Zhang M, Li Z, Hao Y (2013). Clinical pathology research of granulocytic sarcoma of uterine cervix. J Basic Clin Oncol.

[35] Zhang X, Cai X (2013). Partially-differentiated acute myeloblastic leukemia complicating cervical granulocytic sarcoma: report of a case. Chinese Pract J Rural.

[36] Liu X, Li K (2013). Primary granulocytic sarcoma involving oral cavity, mammary gland and uterine cervix: report of a case. Chinese J Clin.

[37] Zhu C, Yang H, Niu J, Zhang Q, Zhu H, Yao Z (2014). Clinical analysis of 15 patients with primary granulocytic sarcoma. J Exp Hematol.

[38] Zuo J, Cheng M, Li Z, Song Y, Wu L (2016). Primary cervical hematopoietic malignancy: a clinicopathologic analysis and literature review. Oncol Prog.

[39] Feng H, Hou L, He L, Jin W, Song X, Liu A (2017). Primary granulocytic sarcoma of cervix: a clinicopathological analysis. Chinese J Diagn Pathol.

[40] Liu X, Shen L, Zhou L, Peng W (2017). Uterine cervical granulocytic sarcoma: report of a case and literature review. Oncoradiology.

[41] Wang Y, Wang H, Zhuang W, Chen H, Zhang C, Li X (2017). Clinical and pathologic features of myeloid sarcoma. J Exp Hematol.

[42] Wang S, Ge L, Zuo D, Zhu D (2005). Uterine solitary granulocytic sarcoma: report of a case. Chinese J Diagn Pathol.

[43] Zhao X, Tang Y, Yan J (2010). Primary granulocytic sarcoma: report of 3 cases and literature review. Shandong Med J.

[44] Hou Z, Shi H, Liang X, Wang X (2012). Granulocytic sarcoma: a clinical and pathologic analysis of ten cases. Chinese J Pathol.

[45] Zhang X, Li A, Li X (2003). Acute leukemia with granulocytic sarcoma as the first manifestation. Clin Misdiagn Misther.

[46] Zheng C, Wu J (2006). Primary granulocytic sarcoma: report of a case and literature review. J Clin Hematol.

[47] Yu H, Ma J, Shi Q, Zhou H, Lu Z (2008). Clinicopathological features of granulocytic sarcoma of the ovary. J Med Postgrad.

[48] Zhou Y, Gao H, Chen Y (2009). Primary granulocytic sarcoma: report of 5 cases. Chinese J Diff Complicated Cases.

[49] Zhou L, Wang M, Ma G (2010). Clinical features and pathology of isolated granulocytic sarcoma of the ovary. Chinese J Postgrad Med.

[50] Zhou D, Huang B (2016). Ovarian granulocytic sarcoma progressing to leukemia: report of a case. J Clin Radiol.

[51] Pang Y, Wan D, Cao W, Zhang S, Chen X, Chen S (2017). Clinical analysis of 57 cases of granulocytic sarcoma. J Basic Clin Oncol.

[52] Zhang L, Liu C (2017). Primary granulocytic sarcoma with vulvar mass as the first presenting symptom: report of a case. Chinese J Pract Gynecol Obstet.

[53] Cheng Z, Xu J, Zou S, Pan H, Zhuang J, Wang W (2004). Granulocytic sarcoma complicating peripheral T-cell lymphoma: report of a case. J Clin Hematol.

[54] Qu Q, Li D, Wang C, Ma L, Li G (2006). Uterine granulocytic sarcoma: report of a case. Chinese Remedies Clin.

[55] Li Y, He A, Lu Y, Lu H (2010). Uterine cervical granulocytic sarcoma: report of a case. Chinese J Pract Gynecol Obstet.

[56] Wu L, He J (2014). Clinical and pathological analysis of granulocytic sarcoma of uterine cervix. Med Inf.

[57] Wang Y, Zhou Y, Luo L, Liu M, Zuo X (2014). Primary granulocytic sarcoma of cervix: a case report and review of the literature. Med J Wuhan Univ.

[58] Long X, Guo Q, Yu X, Qian Y (2012). Granulocytic sarcoma of bilateral ovaries and cervix: report of a case and review of literature. J Diagn Concepts Pract.

[59] Huang D, Wang X, Su G (2013). Analysis on clinical characteristics of 10 patients with granulocytic sarcoma. J Leuk Lymphoma.

[60] Xu J, Deng G (2013). Ovarian and uterine granulocytic sarcoma: report of a case. Guangdong Yixue.

[61] Audouin J, Comperat E, Le Tourneau A, Camilleri-Broet S, Adida C, Molina T (2003). Myeloid sarcoma: clinical and morphologic criteria useful for diagnosis. Int J Surg Pathol.

[62] Muss H, Moloney W (1973). Chloroma and other myeloblastic tumors. Blood.

[63] Byrd J, Edenfield W, Shields D, Dawson N (1995). Extramedullary myeloid cell tumors in acute nonlymphocytic leukemia: a clinical review. J Clin Oncol.

[64] Pathak B, Bruchim I, Brisson M, Hammouda W, Bloom C, Gotlieb W (2006). Granulocytic sarcoma presenting as tumors of the cervix. Digest World Core Med J.

[65] Meis J, Butler J, Osborne B, Manning J (1986). Granulocytic sarcoma in nonleukemic patients. Cancer.

[66] Yamauchi K, Yasuda M (2002). Comparison in treatments of nonleukemic granulocytic sarcoma: report of two cases and a review of 72 cases in the literature. Cancer.

[67] Chevallier P, Mohty M, Lioure B, Michel G, Contentin N, Deconinck E (2008). Allogeneic hematopoietic stem-cell transplantation for myeloid sarcoma: a retrospective study from the SFGM-TC. J Clin Oncol.

[68] Tallman M, Hakimian D, Shaw J, Lissner G, Russell E, Variakojis D (1993). Granulocytic sarcoma is associated with the 8;21 translocation in acute myeloid leukemia. J Clin Oncol.

[69] Paydas S, Zorludemir S, Ergin M (2006). Granulocytic sarcoma: 32 cases and review of the literature. Leuk Lymphoma.

[70] Schafer H, Becker H, Schmitt-Graff A, Lubbert M (2008). Granulocytic sarcoma of Core-binding factor (CBF) acute myeloid leukemia mimicking pancreatic cancer. Leuk Res.

[71] Modi G, Madabhavi I, Panchal H, Patel A, Anand A, Parikh S (2015). Primary vaginal myeloid sarcoma: a rare case report and review of the literature. Case Rep Obstet Gynecol.

[72] Lan T, Lin D, Tien H, Yang R, Chen C, Wu K (2009). Prognostic factors of treatment outcomes in patients with granulocytic sarcoma. Acta Haematol.

